# Mapping methods gaps between EU joint clinical assessments and local health technology assessment decision-making: an environmental scan of guidance in select EU markets and harmonization challenges

**DOI:** 10.57264/cer-2024-0240

**Published:** 2025-02-26

**Authors:** Grammati Sarri, Lydia Vinals, Lilia Leisle, Ingrid Claverie Chau, David Smalbrugge, Kai Lucassen, Yannis Jemiai

**Affiliations:** 1 Cytel Inc., Hamilton House, Mabledon Place, WC1H 9BB, London, UK; 2 Cytel Inc., 1 University Avenue, 3rd Floor, Toronto, ON, M5J 2P1, Canada; 3 Cytel Inc., Potsdamer Strasse 58, 10785 Berlin, Germany; 4 stèveconsultants - a Cytel company, Oullins, France; 5 Cytel Inc., Weena 316-318, Rotterdam, The Netherlands; 6 co.faktor GmbH, a Cytel brand, Potsdamer Strasse 58, 10785 Berlin, Germany; 7 Cytel Inc., 675 Massachusetts Ave, Cambridge, MA 02139, USA

**Keywords:** comparative effectiveness, data, European Union, health technology assessment, joint clinical assessment, methods, uncertainty

## Abstract

**Aim:**

Under the newly instituted health technology assessment (HTA) regulation (HTAR), health technology developers must build evidence packages that meet the needs for both the upcoming EU joint clinical assessment (JCA) and national decision-making. In-depth knowledge of local methodological requirements as well as preparedness for effective strategic development is crucial. This study aimed to review methodological guidance documents to map similarities/misalignments between the EU HTAR and select HTA agencies.

**Materials & methods:**

An environmental scan was performed in March 2024 and updated in December 2024 of the websites for European Network for HTA, the European Commission and HTA agencies in France, Germany, The Netherlands and Spain. The search aimed to systematically identify and summarize methodological guidance documents from the respective organizations on scoping considerations, evidence identification and synthesis.

**Results:**

Overall, published EU HTAR methods guidelines are detailed, prescriptive and make reference to a preference (or lack thereof) for specific analytical methods. There was consensus among EU JCA and local HTA guidelines that clinical comparative assessments should be based on a systematically identified, unbiased selected evidence base derived from various sources. However, agencies differed on guidance related to evidence derived from indirect treatment comparisons.

**Conclusion:**

An environmental scan of methods documents revealed that it will likely be challenging for health technology developers to build strong evidence packages that can support both EU JCA and local reimbursement decision-making. A greater understanding of the similarities and differences between EU and local HTA requirements will be needed, including a greater capacity to demonstrate value through advanced analytics.

## Plain language summary: Methods gaps between EU joint clinical assessments & local health technology assessment decision-making: a review of guidance in select EU markets & harmonization challenges

**What is this article about?:**

A new regulation in the EU aims to provide a standardized way for clinical evidence to support decisions around market access of new health technologies. A comparison of methods standards of EU and local Member States revealed potential challenges for manufacturers to put together evidence that will meet the requirements of all agencies.

**What is the methodology?:**

In this study, guidance documents related to the new EU regulation as well as those for France, Germany, The Netherlands and Spain were reviewed to compare their methods for determining the relative effectiveness of medicines.

**What were the results?:**

There was agreement that evidence on medicines submitted for EU market access should come from the highest-quality sources available. Many differences were identified in what these agencies considered was the best way to handle any data that fell outside of this category.

**Why is this study important?:**

Despite the fact that many new analysis methods exist, they are considered to produce results that are ‘less certain’ and, therefore, their use is viewed with mixed opinions by EU decision-makers. A greater understanding of these similarities and differences will be advantageous for manufacturers in building an evidence package that is suitable for multiple countries.

Access to new health technologies (pharmaceutical and medical devices) in Europe is undergoing an unprecedented transformation. The new EU Regulation 2021/2282 entered in force in January 2022 and was implemented on 12 January 2025 (for oncology and advanced therapy medicinal products). This regulation (and the associated Implementing EU Regulation 2024/1381 [1]) establishes the first legal framework for a unified, EU-centric review process for health technology assessment (HTA), leaving the responsibility of final reimbursement/pricing decisions to local, EU Member States (MS) [2].

HTA is a well-established, systematic and multidisciplinary evaluation procedure that uses explicit methods to determine the value of new health technologies [3,4]. The HTA decision-making process considers direct and indirect consequences for patients, healthcare systems and/or taxpayers with an aim to ‘promote an equitable, efficient and high-quality health system’ [3,4]. The assessment of unmet need, comparative clinical effectiveness, and the financial/budget impact of new technologies is at the core of most HTA frameworks, while many also include assessment of cost–effectiveness and other value drivers (e.g., wider social impact, equity, ethical and legal issues) [5].

The new EU HTA regulation (HTAR) was instituted to address previous concerns around quality and delays in the assessment of new health technologies. It aims to remedy disparities in patient access between MS and create an efficient, standardized EU-wide evaluation process [6,7]. The implementation phase of the HTAR is governed by the HTA Coordination Group, an EU-centralized body, which holds overall responsibility for related procedural and methodological standards. Various implementing acts have provided clarity on the preparatory and operational activities for national authorities, health technology developers (HTD) and other stakeholders [8].

Several activities are central to this implementation phase, including horizon scanning of emerging health technologies, joint scientific consultations (JSC) and joint clinical assessments (JCA) [9]. JSC provides an opportunity for HTDs to gain early scientific advice on the planning and design of clinical studies to meet the expected evidence requirements for a future JCA. A JSC must be requested prior to submission of the final pivotal study protocol to any regulatory authority [10].

JCA, which is the first step of the HTA process, involves the analytical task of gathering, assessing and summarizing available information on benefits and potential harms of new health technologies [11]. The timeline for EU JCA will run in parallel with the European Medicines Agency (EMA) evaluation. The final appraisal step (i.e., value judgement), often described as the political practice of producing guidance and making reimbursement/funding decisions, will remain a national (i.e., EU MS) competency. In the context of the EU HTAR and to meet the needs of all 27 EU MS, JCA is restricted to evidence on the comparative clinical effectiveness and safety of new technologies versus clinical care in the EU. Additional value drivers such as economic, social or other contextual factors will only be considered at the MS level.

Under the HTAR, MS will therefore be required to adapt their existing HTA practices to ensure JCA findings are transparently and consistently incorporated in their local decision-making [12]. This adaptation process will vary by MS depending on the maturity of the HTA system and their level of involvement during the development of the HTAR [13]. For example, Germany and France were influential in shaping the HTAR in terms of establishing processes and methods standards; on the other hand, countries with less-developed HTA systems (e.g., Iceland, Greece and Croatia) must acclimatize to familiarize themselves with the new EU methods standards and adapt how to implement EU JCA findings in local decision-making [14].

The scoping process is a pivotal step in the EU JCA, as it defines the assessment framework by using the population, intervention, comparator, and outcome (PICO) format to define research questions that address the diverse policy needs of EU MS. This standardized approach ensures the assessment reflects local healthcare priorities and guides the data requested from sponsors and evaluated during the JCA.

HTDs face the challenge of generating clinical evidence that meets the needs of the EU JCA, while remaining adaptable to the national HTAs. For this, in-depth knowledge of local methodological requirements as well as a holistic strategy development is required. In this context, the objective of this research was to conduct an environmental scan of methodological guidance documents to highlight and map potential similarities and misalignments between the EU HTAR and local HTA agencies. These documents were adopted as part of the HTAR Implementation Act including previous documents published by the European Network for HTA (EUnetHTA) and selected European HTA agencies in France, Germany, The Netherlands and Spain.

## Materials & methods

An environmental scan was performed in March 2024 and updated in December 2024 of the publicly available websites of EUnetHTA [15], the European Commission [16] and European HTA agencies in France (Haute Autorité de Santé [HAS] [17]), Germany (Institute for Quality and Efficiency in Health Care [IQWiG] [18]), The Netherlands (Zorginstituut Nederland [ZIN] [19]) and Spain (Spanish Agency of Medicines and Medical Devices [AEMPS] [20], Agency for Health Quality and Assessment of Catalonia [AQuAS] [21]). This was selected as the most suitable review approach since the research topic of interest focused on identifying and summarizing methodological guidance documents related to the EU JCA which are available through public websites and not as part of peer-reviewed literature. This type of review was previously considered for similar exercises by HTA bodies [22]. In addition, a mapping exercise was performed to identify trends in similarities and differences across the documents. The scan consisted of a systematic search of the websites from the respective organizations to identify and summarize methodological guidance documents on scoping considerations, evidence identification and synthesis. HTA agencies from France, Germany, The Netherlands and Spain were chosen as high-quality reimbursements systems that were likely to have issued the most comprehensive guidance to date.

A three-step, comparative exercise was conducted to identify themes by matching evidentiary requirements of EU HTAR with the representative, selected HTA agencies. First, the methodological guidance documents of each agency were identified. Second, evidentiary requirements from the EU HTAR were extracted and information was categorized into three areas reflecting the key steps in the JCA implementation: scoping specifications, evidence selection and evidence synthesis. Other specific methodological points for JCA consideration were highlighted. Third, key EU HTAR considerations were qualitatively summarized into statements that were used to map the recommendations from the local HTA methodological guidance documents.

Exclusion criteria included documents that were not endorsed or represented by the selected organizations or that referred to informal HTA processes, HTA-like activities (e.g., consideration of pharmacoeconomic studies on an ad-hoc basis only), and specific activities such as ambulatory care medicines or hospital or digital technologies.

A template was developed specifically to capture information on recommendations for each of the predefined themes; in addition, general information about the type of organization and date of publication was extracted. Website searches and data extraction were conducted independently by two reviewers with a quality check performed by a third, more senior researcher.

## Results

Thirty-one methodological guidance documents were identified, including 15 from EUnetHTA [23–28] and the European Commission [29–37], 15 from the representative HTA agencies (IQWiG [n = 7] [38–44], HAS [n = 5] [45–49], ZIN [n = 2 [50,51]], AEMPS [n = 1] [52]) and one from a local body with a dedicated HTA process (AQuAS [n = 1] [53]).

The documents published by the EU as part of the HTAR replace some of the earlier published documents from EUnetHTA and the latter are not further discussed in this manuscript as these are considered superseded. The scope and the breadth of information provided by each guideline varied considerably, of which the most detailed and comprehensive was from the EU HTAR methods and IQWiG. Eleven of the 16 local methods guidelines were published during the last 5 years.

**Figure 1.**
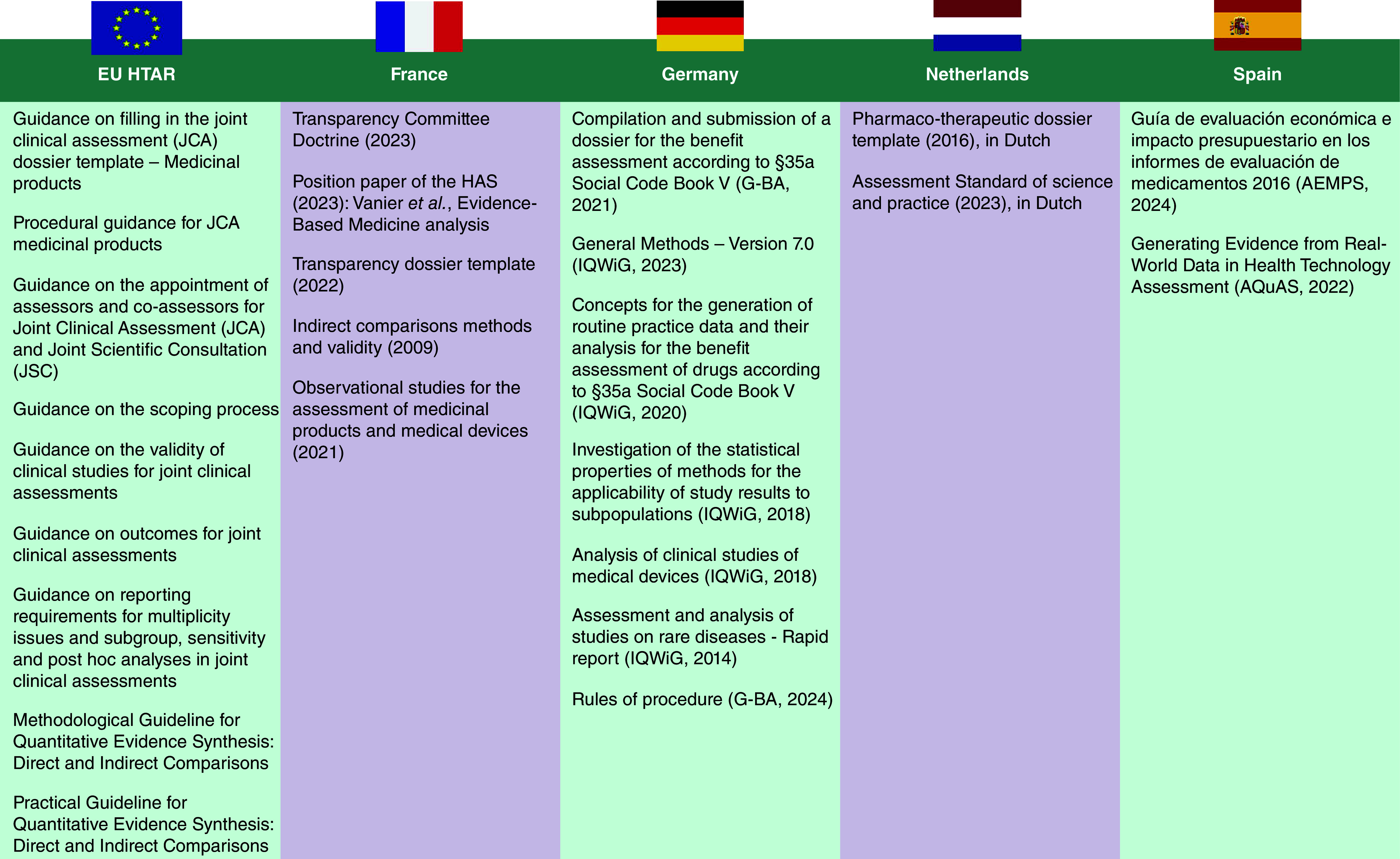
Sources: EU versus French, German, Dutch and Spanish guidance. AEMPS: Spanish Agency of Medicines and Medical Devices; AQuAS: Agency for Health Quality and Assessment of Catalonia; G-BA: Germany’s Federal Joint Committee; HAS: Haute Autorité de Santé; HTAR: Health technology assessment regulation; IQWiG: Institute for Quality and Efficiency in Health Care.

A list of the EU HTAR guidance documents as well as those from France, Germany, The Netherlands and Spain is provided in Figure 1.

### Scoping specifications

A comparison of methodological guidance on scoping specifications between EU JCA and key European HTA agencies is provided in Table 1.

**Table 1.** Commonalities and differences between EU joint clinical assessment methodological guidance and key European health technology assessment bodies: scoping specifications.

EU HTAR	Germany	France	The Netherlands	Spain
Overall assessment scope should follow PICO framework and be inclusive of the needs of all 27 Member States	Only German needs	Only French needs	Only Dutch needs	Only Spanish needs
EMA full label with subgroups of interest should be clearly defined (e.g., delays expected if CHMP opinion makes changes in the submitted indication)	Population does not always align with EMA label	Population does not always align with EMA label	Population does not always align with EMA label	Population does not always align with EMA label
Detailed guidance about defining ‘comparator’ (e.g., off-label treatments, background therapies and individualized treatment comparator)	G-BA defines the comparator	Available local clinical care	SoC (per locally applicable guidelines) or usual care (when no guidelines are available)	Available local clinical care, evidence from public clinical trials and off-label treatments
Equal importance to effectiveness, safety and quality-of-life outcomes	✓	✓	✓	✓

✓ Agreement.

CHMP: Committee for Medicinal Products for Human Use; EMA: European Medicines Agency; G-BA: Germany’s Federal Joint Committee; HTAR: Health technology assessment regulation; PICO: Population, intervention, comparator, outcome; SoC: Standard of care.

Overall, the EU HTAR assessment scope sets up the PICO framework as the cornerstone of the JCA process which, as strongly emphasized, should be inclusive and meet the needs of all 27 EU countries [1]. It is meant to translate national policy questions into research questions and outline the evidence required from the HTD to meet the MS needs [30]. Assessors/co-assessors will draft a PICO survey to be distributed to the MS to define their local needs. A MS can choose not to participate in the survey. Differences are expected between MS regarding populations (national decisions may deviate from the label population submitted to the EMA) and comparators (choice is determined by the local clinical care and can include off-label treatments). The MS will be asked to limit their requests to what is necessary for their national decision-making and specify components of clinical care. The assessors/co-assessors will consolidate the PICO(s), while also considering other relevant input (e.g., from patients and clinical experts). The final evidence requirements (consolidated PICOs) will then be communicated to the HTD.

Since the assessment scope is mostly policy driven, it might contain PICO(s) for which no evidence is available. In this case, the HTD must justify why no data were submitted. In other cases, a specific PICO may not be fully covered by the available studies (e.g., the PICO could comprise only a subgroup of the study population or the requested comparator would not be represented in the study). To meet the evidence requirements, available studies will need to be re-analyzed or assessed for suitability for indirect comparisons. Any deviations from the original study protocols must be clearly mentioned in the JCA dossier, and re-analyses resulting from PICO requests must be provided by the HTD (original study analyses should be included in the dossier regardless) [29].

### Evidence selection

A comparison of methodological guidance on evidence selection between EU JCA and key European HTA agencies is provided in Table 2.

**Table 2.** Commonalities and differences between EU joint clinical assessment methodological guidance and key European health technology assessment bodies: evidence identification and selection.

EU HTAR	Germany	France	The Netherlands	Spain
An SLR with explicit inclusion and exclusion criteria (following PICO framework) is compulsory	✓	Not required	✓	Some details
Database searches (updated 3 months before submission): MEDLINE (e.g., In-Process, other non-indexed citations), CENTRAL, ClinicalTrials.gov, CTIS, EU Clinical Trials Registry, ICTRP, HTA reports from EEA, Australia, Canada, UK, US, JSC recommendations (if available)	General principles (MEDLINE, Embase and CENTRAL, unpublished studies [EU-CTR, CTIS, ICTRP, English and German restrictions), PRESS checklist	✗	No detailed methods; only published literature or EPAR (no conference abstracts)	No detailed methods; only published literature (no conference abstracts)
Study design hierarchy should be considered with RCTs with low RoB as the gold-standard design	✓	✓	✓	✓
Quality assessment of each included study (except for non-RCTs); Cochrane RoB 2 for RCTs and ROBINS-I for comparative non-randomized studies	✓	CONSORT mentioned/Annex 1 information	GRADE	✗
Studies are included in the networks if relevant for a specific PICO unless indirectly contributing to evidence for a given PICO comparison (‘first-order’ loops)	✗	✗	✗	✗
Evidence networks should be conducted at both ‘population’ and ‘comparator’ levels (if different)	✗	✗	✗	✗

✓ Agreement.

✗ Not discussed.

† Health technology developers do not conduct the quality assessment for their original study(ies).

CENTRAL: Cochrane Central Registry of Controlled Trials; CONSORT: CONsolidated Standards of Reporting Trials; CTIS: Clinical Trials Information System; EEA: European Economic Area; EPAR: European public assessment report; EU CTR: European Union Clinical Trial Registry; GRADE: Grading of Recommendations Assessment, Development, and Evaluation; HTA: Health technology assessment; HTAR: Health technology assessment regulation; ICTRP: International Clinical Trials Registry Platform; JSC: Joint scientific consultation; PICO: Population, intervention, comparator, outcome; PRESS: Peer Review of Electronic Search Strategies; RCT: Randomized controlled trial; RoB: Risk of bias; ROBINS-I: Risk Of Bias In Non-randomised Studies - of Interventions; SLR: Systematic literature review.

A systematically identified, comprehensive evidence base constructed from a predefined protocol and clear selection criteria is fundamental to the HTA process. The EU HTAR documents emphasize that PICO requests should guide this process and HTDs should perform a timely, relevant systematic literature review (within three months of dossier submission date) outlining prescriptive requirements for information retrieval (e.g., database searches, patient registries and other sources) and evidence selection scenarios based on the number of comparators, PICOs and network connectedness. Evidence of various types (study designs) and from multiple sources may inform the JCA so systematic searches should consider this in the design phase; to ensure the evidence selection is appropriate and robust for comparative effectiveness analysis, all decisions made in this step must be thoroughly justified and transparently presented. Some differences were noted with local HTA guidelines; for example, a systematic review is not a submission expectation in France whereas in The Netherlands, data sources are only accepted if published in peer-reviewed journals or in the European public assessment report.

Evidence from randomized controlled trials (RCT) retains the ‘gold standard’ position in the evidence hierarchy across the EU methods guidelines considered. In EU HTAR guidance, nonrandomized studies are recommended only to fill evidentiary gaps in situations of unanchored indirect treatment comparisons (ITC) or when this is the only evidence source. However, its use in the JCA context is considered limited. The acceptability of data from nonrandomized studies varied across the other HTA agencies, with stricter requirements set forth by IQWiG compared with the criteria outlined by HAS, ZIN and AEMPs allowing consideration of such evidence in HTAs with high unmet need and challenges in RCT evidence generation (such as rare/very rare diseases, first-in-indication products).

**Figure 2.**
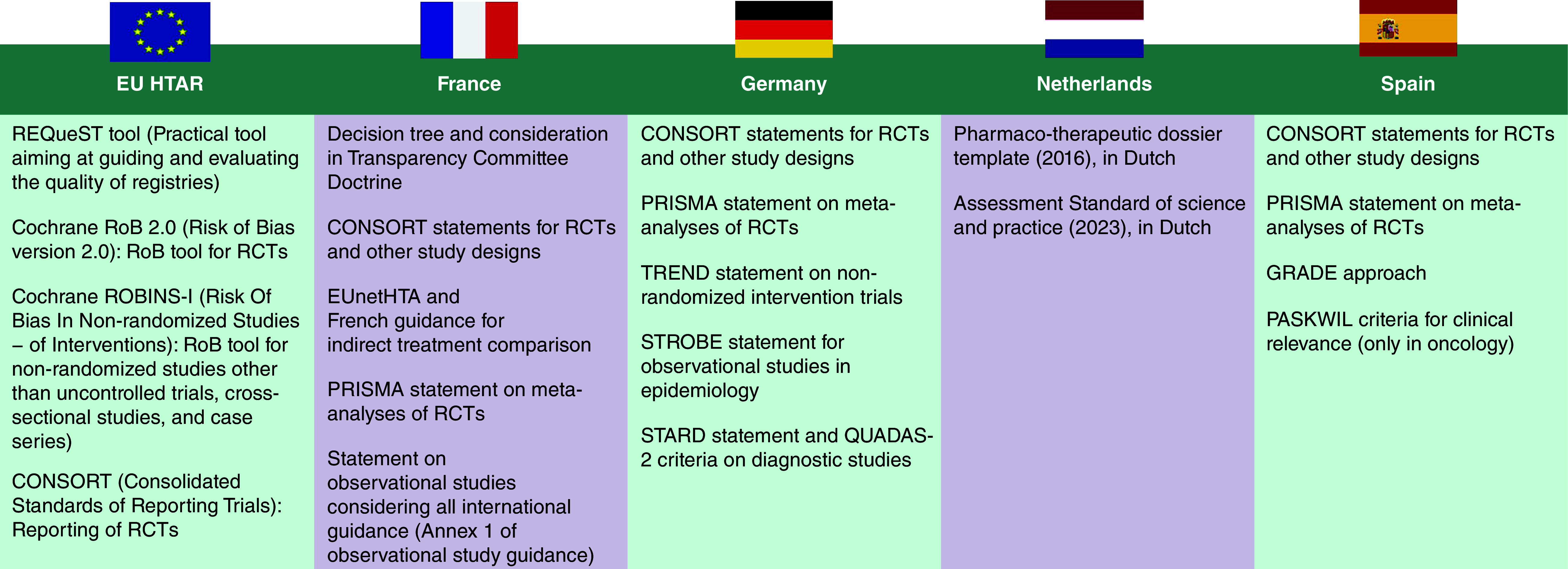
Recommended tools: EU versus French, German, Dutch and Spanish guidance. CONSORT: CONsolidated Standards of Reporting Trials; EUnetHTA: European Network for Health Technology Assessment; GRADE: Grading of Recommendations Assessment, Development, and Evaluation; HTAR: Health technology assessment regulation; PASKWIL: Palliative, adjuvant, specific side effects, quality of life, impact of treatment and level of evidence; PRISMA: Preferred Reporting Items for Systematic reviews and Meta-Analyses; QUADAS: Quality Assessment of Diagnostic Accuracy Studies; RCT: Randomized controlled trial; REQueST: Registry Evaluation and Quality Standards Tool; RoB: Risk of bias; ROBINS-I: Risk Of Bias In Non-randomised Studies - of Interventions; STARD: Standards for Reporting of Diagnostic Accuracy; STROBE: Strengthening the Reporting of Observational Studies in Epidemiology; TREND: Transparent Reporting of Evaluations with Nonrandomized Designs.

One key difference between the EU HTAR and local HTA guidance was the quality assessment step of included studies. Evidence for local submissions must be quality-assessed using the tools recommended by each jurisdiction (Figure 2); for JCA, HTDs must provide a thorough justification and documentation for each quality element and additional contextual information for every study included in the dossier to allow assessors to conduct their own quality assessments.

Other topics covered included the selection criteria for studies to be considered (only if they are directly connected to the evidence networks or contribute as ‘first-order’ loops) for the PICOs set up in the assessment scope. One key difference with the local HTA guidance documents was the EU HTAR request to construct evidence networks at both ‘population’ and ‘comparator’ levels if different, which will have a significant workload impact.

### Evidence synthesis

**Figure 3.**
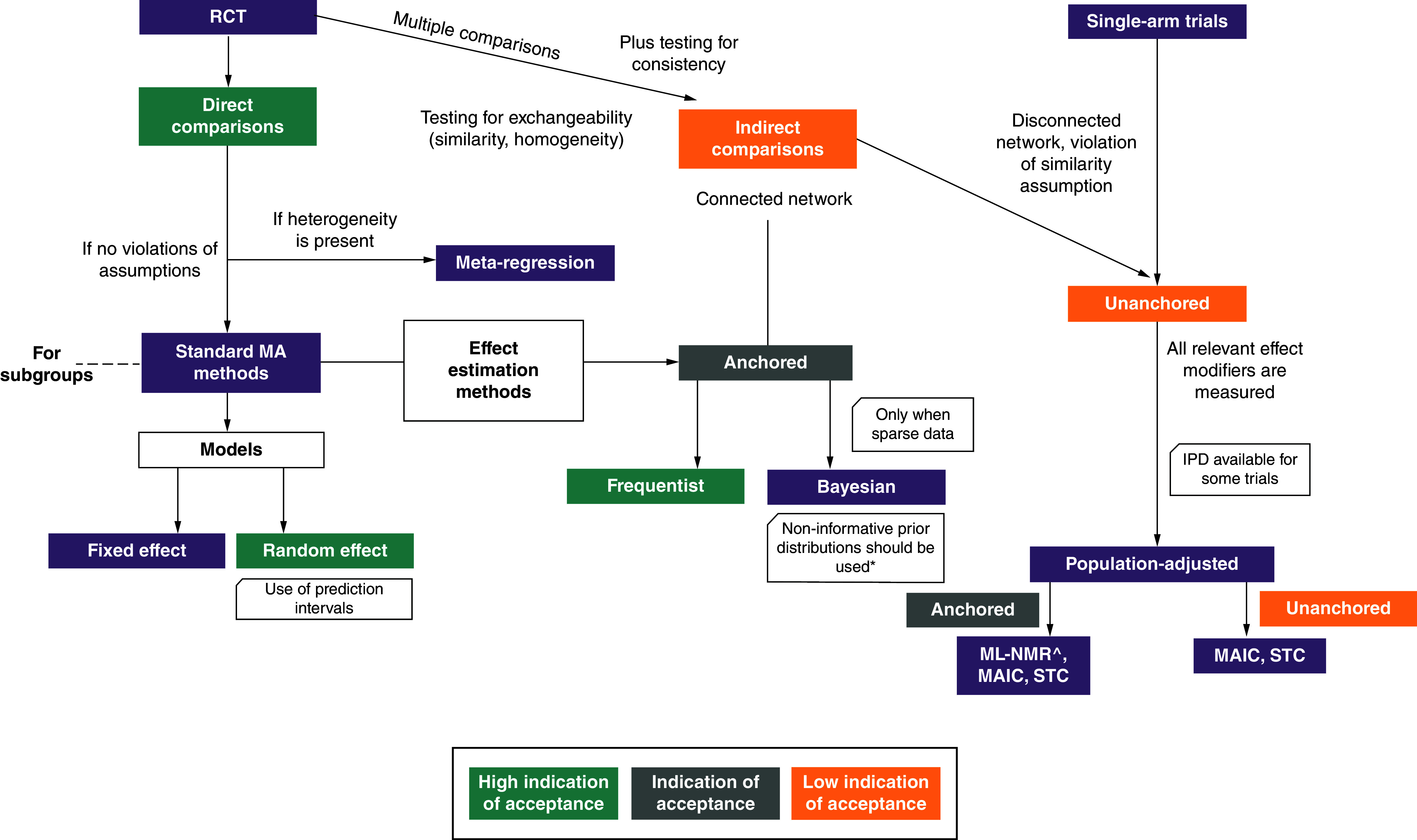
Flowchart evidence synthesis methods as constructed based on EU health technology assessment regulation guidelines. *Informative priors should only be used for the heterogeneity parameter. HTAR: Health technology assessment regulation; IPD: Individual patient data; ITC: Indirect treatment comparison; MA: Meta-analysis; MAIC: Matching-adjusted indirect comparison; ML-NMR: Multilevel, network meta-analysis; RCT: Randomized controlled trial; STC: Simulated treatment comparison.

A comparison of methodological guidance on evidence synthesis between EU JCA and key European HTA agencies is provided in Table 3 and a flowchart constructed based on methodological considerations published as part of the EU HTAR is presented in Figure 3.

**Table 3.** Commonalities and differences between EU joint clinical assessment methodological guidance and key European health technology assessment bodies: evidence synthesis.

EU HTAR	Germany	France	The Netherlands	Spain	Ref.
Feasibility assessment					
Detailed assessment of exchangeability (similarity, homogeneity, consistency [for ITCs]) in each network; when few studies, only similarity can be assessed	✓	Only general principles discussed	Only general principles discussed	Only general principles are discussed	
A priori identification of effect modifiers (literature review, expert input, subgroup analyses); thorough justification and reporting of bias direction from missing effect modifiers	✓	✓	✗	✗	
Quantitative assessment of homogeneity (Q-test, I^2^, forest plot inspection) and inconsistency (Bucher method, deviance and DIC statistics, node-splitting) with full reporting of assumptions and reasoning/ quantitative decision criteria	✓	✗	✗	✗	
Reporting with full reasoning of conclusion regarding exchangeability assessments, pooling of effect estimates across studies and analytical approach selected (including modelling approach, choice of priors and baseline risk adjustments)	✓	Only general principles discussed	✓	✓	
**Comparative analyses**					
A priori definition of JCA SAP is compulsory	✓	✓	✓	✓	
Comparative effectiveness analyses should pool relative treatment effects between treatments and not absolute effects of a particular treatment arm	✓	Clinical relevance is assessed on a case-by-case basis	Distinction between relative outcomes and continuous outcomes	✓	
**Direct comparisons**					
Preference for random-effect models and, in the presence of a connected network, for anchored ITCs (MAIC, STC, ML-NMR) • Population adjustment may not be appropriate when sample sizes are small (difficult to include all relevant effect modifiers)	Detailed guidance (ML-NMR is not included)	Only general principles discussed	✗	✗	
When evidence synthesis is not feasible due to high heterogeneity, use alternative approaches (subgroups splitting [use ICEMAN criteria for credibility assessment], network meta-regression, studies exclusion, PAICs (MAIC, STC, ML-NMR)	Some details (ICEMAN and ML-NMR not included; criteria defined by Sun *et al.*, 2010)	Only general principles discussed	Case-by-case: appropriate justification is needed	✗	[54]
**Indirect comparisons**					
Use of MAIC or STC (for two studies of which one has IPD) and IPD NMR if full IPD network is available	Only adjusted ITCs via common comparators and IPD meta-regression accepted	Only general principles discussed	Case-by-case: appropriate justification is needed	Case-by-case: appropriate justification is needed	
Naive comparisons (i.e., comparisons of absolute outcomes without any adjustment for confounding) should not be used because they do not preserve randomization	✓	✓	Disagreement; naive comparisons are used but downgraded within GRADE system	✗	
**Other topics for consideration**					
Nonrandomized studies (single-arm trials, observational studies) carry a very high risk of confounding bias • Adjustment methods require all effect modifiers and confounders to be measured and prespecified (to be included in the JCA SAP) • Due to greater uncertainty, larger treatment effect sizes are needed	Only in justified exceptional cases; detailed guidance on analytical methods	Detailed guidance on analytical methods (importance of French data use)	Only in justified exceptional cases; minimal guidance on analytical methods	Detailed guide for protocol development (study design, analytics, QC, transparency and replicability)	
Validated surrogate outcomes can be considered but surrogacy should be demonstrated and clearly reported	Stricter criteria	Only if specific context and if with level of proof	More relaxed criteria, can be supported by expert opinion	✗	
**Uncertainty assessments**					
Independently of methods selected, conduct sensitivity analyses and report results in full (assumptions, deviations, directionality)	Additional criteria for sample size by subgroup (≥10) and number of events (≥10)	Considered only as exploratory	✓	✓	
Testing against a shifted null hypothesis for unknown or missing confounders, issues with conditional and marginal effect estimates calculation in STC, distribution of weights in MAIC	Partial agreement	✗	✗	ITCs very unlikely to be acceptable	
Full assessment and description of certainty of results (internal, external validity and statistical precision) is needed; the certainty of results is independent of the medical context of the PICO question	Partial agreement; for extremely rare diseases or very specific disease constellations, the demand for (parallel) comparative studies may be inappropriate	RCT absence can be acceptable based on medical context (unmet need, orphan disease)	Covered within GRADE	✓	
Multiplicity statistical hypothesis is not feasible beyond a few primary and secondary analyses; pre-specification and appropriate results reporting is necessary	✓	✓	Covered within GRADE: downgrade for non specified analyses	✗	
Evidence appraisal systems should not be used; no hierarchy in outcomes assessment	GRADE, inferential statistical thresholds, MCID	MCID	GRADE, MCID	✗	

✓ Agreement.

✗ Not discussed.

DIC: Deviance information criterion; GRADE: Grading of Recommendations Assessment, Development, and Evaluation; HTAR: Health technology assessment regulation; ICEMAN: Instrument for assessing the Credibility of Effect Modification Analyses; IPD: Individual patient data; ITC: Indirect treatment comparison; JCA: Joint clinical assessment; MAIC: Matching-adjusted indirect comparison; MCID: Minimal clinically important difference; ML-NMR: Multilevel network meta-analysis; PAIC: Population-adjusted indirect comparison; PICO: Population, intervention, comparator, outcome; QC: Quality control; RCT: Randomized controlled trial; SAP: Statistical analysis plan; STC: Simulated treatment comparison.

Assessing the clinical effectiveness and safety of the technology compared with routine clinical care (as defined by each PICO) requires quantification of the relative effects of the technology under evaluation. This must be done alongside an assessment of the degree of uncertainty in the relative treatment effects, while factoring in the strengths and limitations of the supporting evidence.

Overall, the EU HTAR methods strongly advocate for the need of direct treatment comparisons (using a frequentist or Bayesian approach) based on high-quality RCTs conducted on the population of interest. Guidance for when to use each approach is provided based on the outcome types, number of available studies and choice of prior distributions (for Bayesian methods). The discussion of analytic methods, however, is preceded by a detailed description of how to assess the exchangeability and heterogeneity assumptions behind each evidence network. Additional considerations for assessing data robustness (missing values, biases), including how this should be quantitatively assessed and presented for the JCA purposes, are also discussed. The EU HTAR methods emphasize the need to identify effect modifiers a prior through multiple, parallel activities (literature review, subgroup analyses, and expert opinion) which should be transparently presented. This may require additional preparatory efforts for HTDs (e.g., conducting targeted literature reviews) which is not considered a routine preparatory activity for local HTA submissions. There is also a reference to the use of the Instrument for assessing the Credibility of Effect Modification Analyses (ICEMAN) [55] which is less applied in national HTAs but provides a structured way to identify credible effect modifications (subgroup effect, statistical interaction, moderation, or heterogeneity of treatment effects) in the dataset. The IQWiG methods guideline does not mention ICEMAN but it discusses the quality and credibility of subgroup analyses, referring to a publication that formulates usability criteria [54].

The EU HTAR guideline (similar to other local HTA standards) requests the full recording of considerations and decisions made in the feasibility assessment of treatment comparisons that will guide the pre-specification of the appropriate analytical approach to be documented in a statistical analysis plan. Regarding analytical methods preferences, naive comparisons of absolute effects (with no confounding adjustment) across different studies should not be used in JCAs since these methods fail to preserve randomization between patients and studies. This was also clearly noted by IQWiG but not in French and Spanish HTA guidelines. ZIN highlighted that such comparisons may be used in local submissions under exceptional circumstances. However, they should be downgraded in the Grading of Recommendations Assessment, Development, and Evaluation (GRADE) assessment due to a preference for direct comparisons analyzed using random-effects models in the case of a connected network. However, the EU HTAR guidelines become less clear when evidence synthesis is needed for indirect comparisons, either due to disconnected networks or in the presence of high heterogeneity. Although the ITC methods (matching-adjusted indirect comparison [MAIC], simulated treatment comparison) and some novel analytical methods (multilevel network meta-regression [ML-NMR; EU HTAR only]) are mentioned, the EU methods clearly spell out situations in which population adjustment may be less valid (e.g., difficulty controlling for all relevant effect modifiers due to small sample size) and inappropriate to use in the JCA context. Even though the JCA guidelines point out that approaches other than ITCs may be explored to answer the PICO questions in these situations (e.g., splitting evidence networks into subgroups, use of meta-regression when appropriate, sensitivity analyses and exclusion of studies), challenges in the implementation and interpretation of comparative effect estimates from these approaches remain for the local HTA purposes. The same concern applies for the use of nonrandomized studies (e.g., single-arm trials and nonrandomized comparative studies) for evidence synthesis; the EU HTAR guidelines specify that this type of data can only be used if there is full access to individual patient data that would allow appropriate use of adjustment methods for all confounders, prognostic variables and effect modifiers to be applied. The EU HTAR methods for these topics are clear and direct; nonrandomized evidence available only at the aggregate level should not be used in the estimation of treatment effectiveness since there are no adequate methods to account for the necessary adjustments. The use of nonrandomized evidence in the German and Dutch HTA context is only allowed in exceptional cases; however, the absolute requirement of access to full individual patient data is less clear than in the EU HTAR guideline. On the other hand, local real-world evidence is considered important for French and Spanish local submissions.

Based on the EU HTAR methods guideline, uncertainty in the relative effects of a technology may be the result of three, mutually independent domains in data analytics: internal validity (albeit systematic error in results due to study design or patient heterogeneity), statistical precision (as a function of random sample variation in results) which should be evaluated under the JCA scope and external validity (generalizability of findings) to be assessed at the local HTA setting. The dichotomization of these uncertainty elements may impair the HTD’s ability to fully explore the impact of some of these considerations on treatment effect estimates and address specific PICO requests that may impose further challenges (e.g., in patient subgroups lacking consideration in the HTD’s primary clinical trial). The EU HTAR methods highlight that sponsors should thoroughly and transparently present uncertainty sources in evidence or analysis considered in the JCA without claiming the medical context of a specific PICO (e.g., rarity of disease) as an explanation for selecting lower-quality evidence or data outside RCTs. This is a clear distinction between the EU and local authorities which allows flexibility in decision-making through their individual access programs for technologies addressing high unmet patient needs or first-in-indication products for rare and very rare diseases, including targeting pediatric populations. Finally, as highlighted in several EU HTAR documents, the scope of JCA is not to provide a value assessment of the technology under review, but rather to provide clinical assessment reports for a range of outcomes that can be used for local, EU decision-making. For that reason, evidence appraisal systems (such as minimal clinically important difference, GRADE and statistical thresholds) and hierarchy in outcomes assessment are not to be used in JCAs. These steps exclusively remain a MS task and some discrepancies in final assessments may be expected due to procedural differences.

Other topics covered included the use of surrogate (or intermediate) outcomes to assess relative effectiveness estimates. In the JCA context, this topic is controversial since the full validation of surrogate outcomes has not been rigorously demonstrated when the submission is completed and may limit the external validity of the submitted evidence. The EU HTAR guidelines specifically point to the fact that a surrogacy validation exercise (testing association between the surrogate and final outcomes) needs to be assessed at the local, MS level but advises local assessors to include all three levels of evidence; the first level is considered a requirement (i.e., correlation of treatment effects between surrogate and final outcomes in the disease stage and interventions as derived from a meta-analysis of several RCTs preferably identified via a systematic literature review). Multiple statistical testing, considerations for sensitivity and subgroup analyses and post-hoc analyses are also discussed in EU HTAR guidance.

## Discussion

A significant number of stakeholders involved in the new EU HTAR expressed concerns about the challenges in the real implementation of this regulation for both local decision-makers and HTDs [56]. Most have voiced concerns around the complexity and intensity of preparatory activities such as how to address multiple PICO requests, the lack of HTD involvement and the tight timelines required to produce meaningful evidence packages to meet the requests of 27 MS [57,58].

Recent discussion, however, has shifted to methodological requirements for evidence synthesis across different data sources and the indirect reference in the HTAR to the acceptability threshold for these analytical methods in the JCA context. According to the European Commissioner for Health and Food Safety, ‘Innovation and technology are key drivers for a strong European Health Union and for bringing medicines to patients’ [59]. Yet, early signs indicate this directive will be difficult to incorporate in the EU JCA process due to limitations on the use of more advanced analytical methodologies that can provide valuable insights on the full spectrum of potential benefits (and harms) of new technologies [60].

Overall, the results from this environmental scan showed that the published EU HTAR methods guidelines are detailed, prescriptive and allude to a preference (or lack thereof) for specific analytical methods. A closer mapping of guidelines, however, revealed intricacies that will make it challenging for HTDs to build strong evidence packages that can support both JCAs and local reimbursement decision-making. For both policy-makers across the EU and HTDs, a deep understanding of the similarities and differences between EU and local HTA requirements will be needed, backed by a greater capacity to demonstrate value through advanced analytical methods. Through EU cooperation, case studies of real-life JCAs will provide the platform to understand the impact on differences in reimbursement decisions across the EU that may be driven by methodological differences at the local level. The exploration of these case studies may further enable discussions in harmonization efforts to ensure alignment in methods standards and benefit patients across the EU.

Methods guidance for the EU JCA most closely aligned with that from IQWiG which was not surprising given the influential role of Germany in the JCA preparation phase [61]. Overall, there was consensus among EU JCA and local HTA guidelines that clinical comparative assessments should be based on a systematically identified, unbiased selected evidence base derived from various sources. A predefined statistical analysis plan, full reporting of exchangeability assumptions and conduct of sensitivity analyses to explore uncertainty are considered alignment points. However, the mapping exercise revealed that the EU HTAR and other local HTA agencies differed on guidance for the appropriate use for ITCs, nonrandomized evidence, or decision criteria for establishing outcomes surrogacy. Lastly, given the JCA scope, evidence appraisals systems or hierarchy outcomes assessment should not be used; instead, this step should be implemented during the local HTA processes.

The use of ITCs in HTA, which is often unavoidable even when RCTs are performed, has been previously debated [62,63]. In the context of EU JCA, the need for ITCs is only expected to increase as HTDs juggle evidence requirements for multiple PICO requests. The EU HTAR outlines concerns about the validity of ITCs in terms of ensuring population homogeneity, confounding effect (and absence of confounders and prognostic variables), missing information, measurement error and data provenance. There was broad consideration by EU HTAR for the use of novel analytical ITC methods (e.g., ML-NMR); however, this was at odds with the strict criteria outlined by the other HTA agencies for unadjusted ITCs and evidence synthesis from nonrandomized studies, putting into question whether these methods are applicable for decision problems at the national level. Different methods estimate distinct types of population-average treatment effects (e.g., MAICs produce marginal treatment effects, whereas ML-NMRs can produce either marginal or population-average conditional treatment effects). This normally does not align in the case of non-collapsible effect measures such as hazard ratios and odds ratios which can lead to various conclusions when comparative findings in JCAs are considered for local decision-making [64,65]. The concern is that meeting the high bar of ITC expectations set up by the EU HTAR guidelines would translate to, in almost every comparative assessment, at least one of these assumptions being violated.

Differences in (and timing of) patient access between European systems due to methodological issues in addition to existing process-related differences is an undesirable situation and at odds with the overall objective of EU HTAR [66]. Differences in local timelines for processing the JCA reports for decision-making are somewhat expected due to current differences in local procedures, availability of local resources and capabilities constraints; Germany and France, for example, have the fastest times to market authorization [67]. However, these differences may pose further risk in increasing existing health disparities between EU countries if variation in methods standards restricts or complicates the opportunity for adaptability of JCA findings to local methods and context in a timely manner [68]. In preparation for the implementation of the EU HTAR, all countries have expressed the need to adapt their local processes to allow for greater flexibility, especially in cases where the JCA does not entirely satisfy the needs of the local health system. However, there is much less direct communication in situations where EU HTAR methods standards do not align with local guidelines. Is it expected that, when EU and local methods guidelines disagree, an indirect endorsement of EU guidance is implied by local HTA bodies? If not, which cases should drive HTD plans of preparing for several, parallel, analytical scenarios, based on the same evidence sources, to meet multiple HTA requirements on different methods standards? These and many other questions are still unresolved as implementation of the EU HTAR is in its infancy.

Taking into consideration these variations, it is important to study the different HTA methods parameters that can influence local acceptance criteria. This knowledge can guide HTDs to develop tailored evidence packages for JCA while keeping in mind the additional expectations at the local HTA level. The mapping of methods differences and similarities across EU JCA and local, MS HTA agencies will also provide clarity for HTDs when assessing the need and impact of mitigation strategies (e.g., multiple analytical scenarios to address the same PICO request using the same evidence base but addressing expectations from various HTA agencies). These methodological variations make JCA processes challenging in their implementation, resulting in different levels of interpretation and potential wide-ranging impact on the local decision-making process and final coverage decisions across MS.

The main limitation of this environmental scan was the selected scope of the individual EU MS and some restrictions around search parameters; therefore, findings from this comparative analysis should be interpreted in this context. The selection focused on MS with higher-quality, more-developed HTA systems that would be expected to produce more complete, methodological guidance than other EU countries with less-developed HTA structures. In addition, some of the conclusions in these observations may be indirectly presented in the reviewed documents without a clear (direct), recommendation-style reference.

Readers are encouraged to use the findings from this environmental scan as a living document and expand the current selection of local HTA agencies to a wider range of MS and settings. The EU HTAR guidelines provide detailed reporting recommendations for each section included in this manuscript; therefore, the current findings should be used alongside these reporting requirements to provide a complete set of EU JCA methods expectations. Local HTA agencies should provide explicit and clear documentation of their methodological considerations in terms of alignment with the EU HTAR guidelines, especially for topics with high uncertainty, to prevent resource waste (for both HTDs and assessors) and make reports publicly available when interpreting JCA findings for their local decision-making. That will not only provide justification for any deviations between MS in the benefit assessments of health technologies but will create a repository (reference point) for future JCA submissions for all involved in the HTA at the EU level. While entering the first phase of EU HTAR implementation, it is crucial to monitor the real-life extrapolation of these methods standards in the JCA process by identifying opportunities for methods updates and further research investment.

## Conclusion

The new EU HTAR aims to revolutionize the timely access of innovative health technologies for EU patients by centralizing the clinical comparative assessment of the HTA process, allowing other HTA aspects, pricing negotiations and final reimbursement decisions at the local, EU MS level. This comparative mapping of EU methodological documents published under this new regulation and the local HTA guidelines revealed several methodological differences with the closest alignment found between EU HTAR and IQWiG. HTDs need to anticipate how these differences may affect decisions when developing evidence generation plans for the EU JCA submission of their products and anticipate the need for adjusting value stories for local HTA submissions. Closer methodological harmonization between local European HTAs and EU HTAR would ensure smooth transferability of EU JCA to local settings without duplicated efforts for all stakeholders involved (HTDs, EU assessors and local decision-makers) and make progress toward the fundamental principal of EU health equity which was the impetus for the institution of this new EU HTAR.

## Summary points

- The new EU Regulation 2021/2282 will establish a legal framework for a unified, EU-centric review process of clinical comparative assessment for new health technologies undergoing a marketing authorization request process in the EU. This regulation aims to address previous concerns around quality and delays in the assessment of new health technologies.
- Other steps in the health technology assessment (HTA) process, such as cost and budget assessments, pricing negotiations and final reimbursement decisions will remain the responsibility of EU Member States.
- Scoping of the population, intervention, comparator and outcome considerations and joint clinical assessment (JCA) are the steps of this new regulation. JCA involves the analytical task of gathering, assessing and summarizing available information on benefits and potential harms of new health technologies.
- Health technology developers will need to build evidence packages for new medicines that meet the requirements of both EU HTA regulation (HTAR) and multiple HTA agencies to be submitted in parallel with the market authorization process.
- Methods documents for the EU HTAR, as well as those for France, Germany, The Netherlands and Spain were reviewed, followed by a mapping exercise to identify similarities/differences across the guidance.
- Overall, EU HTAR methods guidelines contain comprehensive details of different aspects of scoping requirements, evidence identification and synthesis process and provide specific analytical recommendations.
- There was consensus among EU JCA and local HTA guidelines that clinical comparative assessments should be based on predefined statistical analysis protocols, using systematically identified, high-quality evidence, preferably from randomized controlled trials and using direct comparative effectiveness methods.
- The acceptance (or indication of acceptance) of evidence derived from indirect treatment comparisons, especially in unanchored treatment comparison situations and using evidence outside clinical trials, varied across the guidance documents. Differences were also noted in terms of detailed reference and methods for exploring data uncertainty in comparative effect estimates for technologies under assessment.
- A greater knowledge base and critical impact assessment on the similarities/differences across methods guidance will help health technology developers build strong evidence packages that can support both EU JCA and local reimbursement decision-making.
